# When autophagy meets placenta development and pregnancy complications

**DOI:** 10.3389/fcell.2024.1327167

**Published:** 2024-02-02

**Authors:** Pei Zhou, Junqi Wang, Jun Wang, Xiaomei Liu

**Affiliations:** ^1^ Department of Obstetrics and Gynecology, Shengjing Hospital of China Medical University, Shenyang, Liaoning, China; ^2^ Department of Obstetrics and Gynecology, Benxi Central Hospital of China Medical University, Benxi, Liaoning, China

**Keywords:** autophagy, placenta, pregnancy complications, gestational hypertension (GH), intrauterine growth restriction (IUGR)

## Abstract

Autophagy is a common biological phenomenon in eukaryotes that has evolved and reshaped to maintain cellular homeostasis. Under the pressure of starvation, hypoxia, and immune damage, autophagy provides energy and nutrients to cells, which benefits cell survival. In mammals, autophagy is an early embryonic nutrient supply system involved in early embryonic development, implantation, and pregnancy maintenance. Recent studies have found that autophagy imbalance in placental tissue plays a key role in the occurrence and development of pregnancy complications, such as gestational hypertension, gestational obesity, premature birth, miscarriage, and intrauterine growth restriction. This mini-review summarizes the molecular mechanism of autophagy regulation, the autophagy pathways, and related factors involved in placental tissue and comprehensively describes the role of autophagy in pregnancy complications.

## 1 Introduction

The placenta plays a crucial role in the nutrient exchange and energy supply between the mother and the fetus to meet their respective demands for nutrients and energy during pregnancy (Musa et al., 2023). Compared to non-pregnant women, pregnant women have greater nutrient requirements and higher energy expenditure, such as during the rapid fetal growth phase or under starvation, hypoxia, and malnutrition (Marshall et al., 2022). Accordingly, placental metabolism undergoes adjustments to adapt to such stressful changes. Studies have shown that incomplete placental development or dysfunction affects embryo implantation, fetal nutrition, and energy supply and influences pregnancy outcomes and neonatal health (Knöfler et al., 2019). Thus, the placenta plays an important role in maintaining the normal physiological activity of pregnant women and fetuses. Autophagy is an evolutionarily conserved mechanism. Autophagosomes were first observed under an electron microscope in 1962 (Ashford and Porter, 1962). The classic example of autophagy activation was observed in yeast during the 1990s, along with the related genes (Takeshige et al., 1992). At the physiological level, autophagy has been recognized as an essential protective mechanism that helps maintain organismal stability and resist external dangers (Luo et al., 2022). Autophagy research has attracted increasing research attention and interest.

In 2008, it was reported that autophagy-related proteins such as Beclin-1, microtubule-associated protein 1A/1B-light chain 3 (LC3B), and DRAM were present in the human placenta, and increased autophagic activity in the placenta may be implicated in the pathophysiology of pre-eclampsia (PE) (Oh et al., 2008). Autophagy is involved in the development of the human placenta, and changes in oxygen and glucose levels participate in the regulation of autophagic changes in cytotrophoblast cells (Zhao et al., 2021). The specific mechanism is due to excessive accumulation of P62 in the placenta, which leads to a decrease in placental growth factor levels, inhibition of the invasive function of trophoblast cells, increased apoptosis, insufficient invasion of the shallow layer of the trophoblast and inadequate vascular remodeling, which is an important pathological basis for the occurrence of pre-eclampsia (Aoki et al., 2018). Therefore, autophagy dysfunction is closely related to various pregnancy complications. This review provides a concise overview of the currently known molecular mechanism governing autophagy regulation, describes the pathways and regulatory factors implicated in autophagy within placental tissue, and comprehensively summarizes the significance of autophagy in pregnancy complications. Furthermore, it offers novel therapeutic targets for addressing pregnancy-related disorders associated with dysregulated autophagy.

## 2 Autophagy and its classification

### 2.1 Autophagy and basic pathway

**FIGURE 1:**
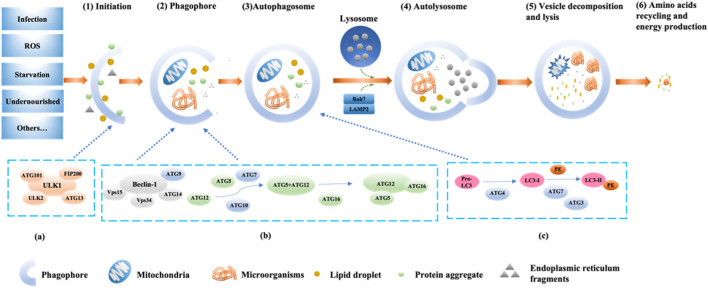
The pathway and mechanism of autophagy (Initiation→ Phagophore→Autophagosome→Autolysosome→Vesicle decomposition and lysis→Amino acids recycling and energy production). When the cell receives a nutrient-rich autophagy-inducing signal, mTOR can be activated and inhibit various autophagy regulatory complexes such as ULK1/2, ATG13, IP200, and ATG101 **(A)**; conversely, mTOR is inhibited under conditions of nutrient deficiency, starvation, *etc.*, thereby activating autophagy. Subsequently, the Beclin1-ATG14L-Vps15-Vps34 complex is activated, promoting the formation of phagophores. With the assistance of ATG7 and ATG10, ATG12 conjugates to ATG5, then binds with ATG16 and completes polymerization to form the ATG12-ATG5-ATG16L complex **(B)**, which can promote LC3-I lipidation into LC3-II. Additionally, under the continuous action of ATG4, ATG7, and ATG3, PE binds with LC3-II **(C)**. Subsequently, LC3-II is inserted into the autophagosomes. The autophagosome forms an autolysosome with the assistance of LAMP2 and Rab7. Finally, engulfed proteins or organelles are degraded within the autolysosome, and LC3B-II is also degraded and recycled.

Autophagy is a process of programmed protein degradation in eukaryotic organisms, under the action of autophagy-related genes, to maintain the cellular environment by degrading long-lived proteins, damaged organelles, and harmful substances (Mizushima and Komatsu, 2011). Starvation, nutrient deficiency, and rapamycin can activate autophagy (Wang and Chen, 2022). Yun et al. confirmed that under the condition of nutrient deficiency, the excessive reactive oxygen species (ROS) produced by energy stress in the body and H_2_O_2_ produced by mitochondria through Cys81 sulfhydryl modification promote the cleavage of newly synthesized LC3B by the reduced form of ATG4, inducing the formation of autophagosome (Yun et al., 2020). Inducing factors for cellular autophagy include immune signals, mitochondrial damage, oxidative stress, and endoplasmic reticulum stress (ERS) (Figure 1). Therefore, upstream factors that activate autophagy include Beclin-1, ATG1/unc-51, PI3K, VMP1, ATG12, ATG8, ATG5, ATG7, and other factors (Kroemer et al., 2010; Kang et al., 2011; Levine and Kroemer, 2019). The main functions of autophagy-related genes (ATG) include promoting abnormal protein targeting transport to lysosomes, coordinating the production and maturation of autophagosomes, participating in various cellular pathways, assisting intracellular material transmembrane transport (Levine and Kroemer, 2019; Staiano and Zappa, 2019). In 1993, Staiano and others reported that about 40 autophagy-related genes were involved in the activation mechanism of autophagy, and the first 15 ATG genes were determined through genetic screening of yeast (Staiano and Zappa, 2019; Huang et al., 2020).

### 2.2 Autophagy regulation mechanism

Currently, the known major regulatory pathways for autophagy include PI3K-AKT-mammalian target of rapamycin (mTOR), NFκB/Bcl-2, and MAPK pathways (Jacomin et al., 2018; Huang et al., 2020; Huang et al., 2021). The currently known core complex of autophagy includes 1) ULK1 protein kinase complex; 2) Beclin-1-Vps34 lipid kinase complex; 3) Atg9-WIPI-1 complex; 4) Atg12 conjugation system; 5) LC3 lipidation system (Liu et al., 2023). The process of autophagy involves the initiation, nucleation, and expansion of autophagic membranes (Figure 1) (Bodineau et al., 2022). The key regulatory point for autophagy in mammals is the protein mTOR (Rabanal-Ruiz et al., 2017). The PI3K-AKT-mTOR pathway is the classical pathway for autophagy regulation. mTOR belongs to the PI3K-related protein kinase family, and the C-terminal of mTOR is homologous to PI3K. mTOR has two forms of complexes, mTORC1 (lysosomal mTOR) and mTORC2 (membrane mTOR). mTORC1 mainly participates in autophagosome regulation, while mTORC2 mainly participates in cell skeleton regulation (Sun et al., 2020). When the body is deficient in nutrients, the activity of mTOR is inhibited, and the Ser757 phosphorylation site on mTOR dissociates from ULK1 through dephosphorylation. Subsequently, the Ser317 and Ser777 sites of ULK1 are phosphorylated and activated by AMP-activated protein kinase, promoting the formation of the ULK1 complex (ULK1, ULK2, FIP200, ATG101, ATG13). Then, phosphorylation of phosphatidylinositol (PI) by PI3K produces phosphatidylinositol triphosphate (PI3P), which is a key membrane marker for autophagosome formation in cells. It can be activated by binding to serine/threonine protein kinase Vps15 and then bind to Beclin-1 phosphorylated and activated by ULK1 to form the PI3K-Vps15-Beclin-1 complex, inducing vesicle nucleation (Bae et al., 2017; Noda, 2017; Chung et al., 2019; Xiang et al., 2020). Finally, ATG4B, ATG3, ATG7, ATG10, ATG16, and other factors promote the binding of microtubule-associated protein 1 light chain 3 (LC3) to phosphatidylethanolamine, promoting the transformation of LC3-I into LC3-II, participating in the extension and closure of autophagic vesicles membrane (Figure 1) (Sato et al., 2019). Subsequently, the double-layer structure of the autophagic vesicles continuously elongates and wraps the substrates that need to be degraded to form autophagosomes, and the autophagosomes fuse with the lysosomes to form autophagolysosomes, which further facilitates the degradation of the coated substrate(s) of the coated substrate. Finally, the degradation products (amino acids, nucleotides, *etc.*) can be recycled by the body, and the residue is expelled from the body (Mizushima and Komatsu, 2011; Li J. et al., 2020). In addition, further research has shown that UV radiation resistance-related gene (UVRAG) can enhance the interaction between BECN1 and PIK3 and increase PtdIns3K activity by binding to BECN1, thereby further promoting autophagosome formation (Smith and Wilkinson, 2017).

### 2.3 Autophagy classification

According to the different ways in which substrates enter lysosomes during the activation process and whether double-membrane vesicles are formed, autophagy is divided into three types: macroautophagy, chaperone-mediated autophagy (CMA), and microautophagy (Gomes et al., 2017). Macroautophagy is a non-selective degradation pathway that is the most characterized, which forms autophagosomes that engulf materials in the cell and then transport them to lysosomes along microtubules. The materials are degraded after fusion with the lysosomal outer membrane (Hayashi et al., 2020). Microautophagy is characterized by the direct invagination or protrusion of a lysosomal or vacuolar membrane, which then engulfs the cell content (Kuchitsu et al., 2023). CMA is highly selective and involves chaperone molecules such as the carboxyl terminus of HSC70-interacting protein (CHIP) and heat shock protein 40 (HSP40). Substrates in the cell are recognized by these molecules and then transported to lysosomes for degradation. For example, HSC70 recognizes the KFERQ motif and binds to lysosome-associated membrane protein 2A (LAMP2A) to promote degradation (Kaushik and Cuervo, 2018).

Autophagy can also be classified according to the degraded organelle (Vargas et al., 2023). Endoplasmic reticulum autophagy is a selective autophagy pathway that compensates for restoring endoplasmic reticulum function. When cells are subjected to external stimulation such as nutrient deficiency, metabolic imbalance, and oxidative stress, ERS signaling pathways such as sterol regulation reaction, endoplasmic reticulum to overload response (EOR), and unfolded protein response (UPR) are activated, initiating self-protective mechanisms to maintain normal endoplasmic reticulum function by clearing accumulated unfolded and misfolded proteins (Liang et al., 2018; Qi and Chen, 2019).

Recent studies have shown that the interaction between ERS and autophagy is beneficial for restoring endoplasmic reticulum function (Ogata et al., 2006; Fujita et al., 2007; Liu et al., 2010; Féral et al., 2021). Endoplasmic reticulum autophagy mainly occurs near the phospholipase-rich subregion of the endoplasmic reticulum, and the PERK, IRE1, and ATF6 pathways that regulate UPR are involved in endoplasmic reticulum autophagy. PERK is activated by autophosphorylation and promotes Atg12 expression by phosphorylating eIFα, leading to LC3 I conversion to LC3 II, which further activates autophagy. IRE1 forms a complex with tumor necrosis factor receptor-associated factor-2 (TRAF-2), which phosphorylates apoptosis signal-regulating kinase 1 (ASK1). Phosphorylated ASK1 can induce JNK phosphorylation to activate Bcl-2 and initiate cellular autophagy. ATF6 comprises ATF6α and ATF6β, but only ATF6α plays a major role in the ATF-dependent transduction of UPR signals. ATF6α can activate autophagy by regulating the transcriptional levels of XBP1 and CHOP.

Mitophagy is also a selective autophagy. Mitochondria, an important double nuclear organelle in mammalian cells, is also an important site for aerobic respiration and ATP production (Annesley and Fisher, 2019). Mitophagy can be classified into ubiquitin-dependent mitophagy and non-ubiquitin-dependent mitophagy based on different mechanisms (Ma et al., 2023). In mammals, the most characteristic ubiquitination-dependent mitophagy mechanism is PINK1/Parkin. PINK1/Parkin accumulates heavily in damaged mitochondria and undergoes self-oxidative phosphorylation. The oxidized and phosphorylated PINK1/Parkin can activate the E3 ligase activity of Parkin, which leads to the ubiquitination of the outer mitochondrial membrane and binding to LC3B, thereby degrading damaged mitochondria (Furuya, 2018). In addition, non-Parkin-dependent degradation autophagy is another ubiquitin-dependent autophagy, mainly regulated by Gp78, Smad ubiquitination regulatory factor-1 (SMURF1), autophagy and Beclin 1 regulator (AMBRA1) (Villa et al., 2018). Non-ubiquitin-dependent mitophagy refers to the direct binding of mitochondrial proteins to LC3, thereby degrading damaged mitochondria. Its related autophagy receptors include FUN4 domain-containing protein 1 (FUNDC1), BCL.2/adenovirus E1B 19kD-interacting protein 3 (BNIP3), BCL2-like protein 13 (BCL2L13), FK506-binding protein 13 (FKBP13), Prohibin 2 (PHB2), Nip3-like protein X (NIX) and cardiolipin (Chen et al., 2016; Marinković et al., 2021).

Peroxisomal autophagy is also a type of selective autophagy. Peroxisomes are widely present metabolic organelles in eukaryotic cells (Wilhelm et al., 2022). They mainly participate in cellular redox balance and lipid metabolism, including fatty acid beta-oxidation and ether phospholipid synthesis (Demers et al., 2023)^.^ Multiple studies have shown that human metabolic disorders can lead to peroxisomal dysfunction and induce many metabolic diseases (Stradomska, 2018). In mammals, peroxisomal autophagy is mainly induced by Lon protease and 15-lipoxygenase-expressing protein released from the cytoplasm for degradation. Autophagy genes involved include ATG30, ATG37, ATG17, ATG28 (Luo and Zhuang, 2018). Additionally, studies have found that excessive accumulation of ROS can activate peroxisomal autophagy and induce degradation (Shim et al., 2023).

In addition to the traditional forms of autophagy mentioned above, several new selective autophagy have been discovered in recent years, including Ribophagy and Proteaphagy. Ribophagy selective degradation of ribosomes through autophagy. Inhibition of mTOR, starvation, and arsenic treatment can induce ribophagy (An and Harper, 2018). Kraft et al. identified two proteins, ubiquitin-specific protease 3 (Ubp3) and Ubp3-associated factor (Bres), that are involved in the selective degradation of ribosomes but not in macroautophagy (Kraft and Peter, 2008). N Nuclear FMR1-​interacting protein 1 (NUFIP1) has been shown to function as a receptor for ribophagy in mammals. NUFIP1 directly interacts with LC3B and ribosomes, promoting ribophagy, while the reduction of NUFIP1 inhibits ribophagy (Wyant et al., 2018). Proteaphagy is the selective degradation of proteasomes through autophagy. In mammalian cells, the main process of proteaphagy involves the recognition of ubiquitinated proteasomes by the autophagy receptor p62, which interacts with the ATG8 protein tethered to the autophagosome membrane. Fusion with lysosomes ensures enzymatic degradation of captured proteasomes (Quinet et al., 2020). Currently, there are no reports linking ribophagy or proteaphagy to placental development and pregnancy complications.

Unlike physiological autophagy, excessive autophagy can mediate programmed cell death—autosis, whose main characteristic is the large-scale cytoplasmic vacuolization in dying cells. Because it differs from other forms of cell death, autophagy-mediated cell death (autophagic cell death) is also known as type II programmed cell death (Nah et al., 2020). Kang et al. have found that autosis recruits Atg proteins, Beclin-1 regulatory factor (AMBRA1), Ultraviolet resistance-associated gene (UVRAG), and Rubicon to the nucleation site via Beclin-1, regulates the lipid kinase activity of the PI3K complex, promotes the formation of the Beclin 1-Vps34-Vps15 core complex, and thereby regulates the occurrence of autophagy. Dysregulation of these proteins will lead to autosis (Kang et al., 2011). In recent years, studies have indicated that autosis also plays a crucial role in maintaining the function of biological cells and the development of diseases. The following conditions must be met to define autosis: 1) Other causes of cell death must be ruled out; 2) Autophagic flux increased in cells; 3) Genetic or pharmacological inhibition of autophagy can rescue cells (Schwartz, 2021).

In conclusion, the regulation of autophagy is diverse and complex, and its specific mechanisms are yet to be explored. This article mainly elaborates on the regulation of autophagy-related pregnancy complications.

## 3 Autophagy in placenta

The placenta is a vital organ between the mother and fetus, with functions such as substance exchange, energy supply, immunity, and secretion (Adibi et al., 2021). The normal structure of the placenta represents the physical structure of nutrient exchange, which is crucial for maintaining fetal growth. Therefore, changes in the placenta’s morphology, structure, and function can lead to diseases (Burton et al., 2016). In recent years, investigators have found that dysregulation of autophagic function in the placenta can change placental structure and function and participate in the occurrence and development of various pregnancy complications (Oh and Roh, 2017). For example, through different pathways, malnutrition, ER stress, hypoxia, and oxidative stress can activate autophagy in the placenta during pregnancy. Dysregulation of autophagy in placental tissue is closely related to pregnancy complications such as premature delivery, miscarriage, intrauterine growth restriction, pregnancy-induced hypertension, gestational diabetes, and gestational obesity (Oh and Roh, 2017).

Interestingly, Huang et al. found that LC3B expression was reduced, and the autophagy receptor SQSTM1/p62 expression was significantly increased in the testes of male mice after knocking out ATG5, leading to a decrease in sperm quantity and motility, which resulted in a decline in male fertility (Huang et al., 2021). Therefore, autophagy plays a crucial role not only in female fertility but also in male fertility. In the following text, we use two tables to summarize the autophagy-related molecules that play important roles in pregnancy complications (Table 1) and make a summary of available studies associated with deregulated autophagy in pregnancy complications (Table 2). This review aims to further elaborate on the role of autophagic dysregulation in the placenta concerning pregnancy complications and may provide new insights into treating these diseases.

**TABLE 1:** Summary of autophagy-related molecules in pregnancy complications.

Autophagy-related molecules	Participating in the autophagy process	Functions	Associated pregnancy complications	References
mTOR	Initiation of autophagy	Regulate cell growth and perceive fluctuations in nutrient levels	Miscarriage	Wei et al. (2020), Hong et al. (2021), Lu et al. (2021)
			IUGR	Dai et al. (2021)
			Gestational Obesity	Kelly et al. (2020)
ULK1	Initiation of autophagy	Regulate the formation and transportation of autophagic vesicles	Gestational Obesity	Upadhyay et al. (2019)
PI3K	Autophagosome maturation	Participate in cell proliferation, differentiation, apoptosis, and glucose transport		
Beclin-1	Autophagosome maturation	Participate in autophagy, cell apoptosis, and immune regulation	Miscarriage	Hong et al. (2021)
			IUGR	Dai et al. (2021), Wang et al. (2021)
			Gestational hypertension	Liu et al. (2022)
			GDM	Avagliano et al. (2017b), Hung et al. (2020), Luo et al. (2020)
LC3	Autophagosome maturation	Participate in autophagosome formation	Miscarriage	Hong et al. (2021)
			Premature Birth	Cao et al. (2016), Avagliano et al. (2017a)
			IUGR	Dai et al. (2021), Wang et al. (2021), Zhu et al. (2021)
			Gestational hypertension	Lee et al. (2021), Liu et al. (2022)
			GDM	Avagliano et al. (2017b), Li et al. (2020b), Hung et al. (2020), Wang et al. (2020)
			Gestational Obesity	Upadhyay et al. (2019)
Vps15	Autophagosome maturation	Participate in intracellular autophagosome formation		
ATGs	Autophagosome maturation	Regulate microtubule and autophagosome formation	Miscarriage	Hong et al. (2021)
			Premature Birth	Cao et al. (2016)
			Gestational hypertension GDM	Sharma (2018), Lee et al. (2021)
			Gestational Obesity	Li et al. (2020b), Hung et al. (2020), Wang et al. (2020)
				Upadhyay et al. (2019)

**TABLE 2:** Summary of available studies associated with deregulated autophagy in pregnancy complications.

Study	Objects	Tissue/cells	Autophagic alterations and effects
Tan et al. (2020)	Human	Placental decidua and villi, dNK cell, HTR-8/Sneo cell	Impaired autophagy in trophoblasts may cause miscarriage through activating decidual NK cytotoxicity
Hong et al. (2021)	Human	Endometrium、HESC cell	Enhanced autophagy in the endometrium promotes recurrent miscarriage due to the crosstalk with amino acid metabolism
Pan et al. (2021)	Human	Placental villous trophoblast cell, JAR cell	Aberrant-activated autophagy in cytotrophoblasts is related to recurrent miscarriage
Avagliano et al. (2017a)	Human	Placental villous trophoblast cell	Autophagy enhancement in inflammatory placenta is a sign of preterm birth
Cao et al. (2016)	Human and mice	Placenta trophoblast cell	Autophagy deficiency in syncytial trophoblasts may lead to preterm birth
Zhu et al. (2021)	Human and mice	Placenta, JEG-3 cell	Cadmium-induced excessive mitophagy may be one reason for FGR
Dai et al. (2021)	Human	Placenta、BeWo cell	Asymmetric dimethylarginine (ADMA)-induced autophagy in trophoblasts may cause FGR.
Sharma (2018)	Mice	Placenta	Increased autophagy may be a diagnostic method for pregnancy hypertension and pre-eclampsia
Lee et al. (2021)	Mice	Uterus	Autophagic deficit in the uterine could assess the status of pre-eclampsia
Avagliano et al. (2017b)	Human	Placenta, fetus	Autophagy enhancement in the placental may be related to GDM.
Wang et al. (2020)	Human	Placenta, HTR8/SVneo cell	Upregulated autophagy in the placenta of extravillous trophoblast cells under high-glucose conditions could promote gestational diabetes mellitus (GDM)
Li et al. (2020b)	Human	Placenta	Enhanced autophagy in the placenta may be related to GDM
Hung et al. (2020)	Human	Placenta, Primary Human Trophoblast	Decreased autophagy in GDM patients might be associated with the delivery of large-for-gestational-age infants
Simon et al. (2017)	Human	Placenta, Primary Human Trophoblast	Autophagy formation and autophagy activation in placental villous trophoblast cells may be related to Gestational Obesity
Upadhyay et al. (2019)	Mice	Placenta	Restoring autophagy under time-restricted feeding conditions may block HFD-induced placental apoptosis and inflammation
Diceglie et al. (2021)	Human	Placenta	Upregulation of autophagy may be related to maternal obesity and GDM

## 4 Autophagy-related pregnancy complications

### 4.1 The role of autophagy in miscarriage

The causes of miscarriage in women are diverse, including immunological factors, self-chromosome factors, genetic factors, and uterine malformations, but many potential pathogenic mechanisms are still unknown (Haas et al., 2019). In recent years, it has been found that female miscarriage may be closely related to the dysfunction of placental autophagy.

Tan et al. found that there were autophagy inhibition in the trophoblasts of recurrent spontaneous abortion (RSA) patients, which may increase the toxicity of decidua NK cells and lead to miscarriage through the targeted destruction of the trophoblast invasion by IGF-2 and PEG10 genes (Tan et al., 2020). Further investigation with electron microscopy revealed that the autophagy level in villi of the recurrent miscarriage (RM) is lower compared to the normal population. Second, by treating dNK cells and HTR-8/Svneo cells with 3-MA, rapamycin, IGF-2, and other methods *in vitro*, it was confirmed that the invasion of HTR-8/Sneo cells decreased significantly after treatment with 3-MA, and the toxicity ability of NK cells increased. Rapamycin can inhibit the toxicity of NK cells by restoring the autophagy level of nutrient cells. Interestingly, supplementing the NK cell with the functional gene IGF-2 can partially reverse the autophagy activation effect of rapamycin. After the autophagy inhibition of the trophoblast cells occurs, the level of PEG10, an invasion-related gene of the trophoblast, also decreases. *In vivo* experimental results also showed that the level of IGF-2 in the villi of RSA patients was higher than that of normal pregnant women, and the expression of PEG10 in the villi of RSA patients was lower than that in normal pregnant women.

Hong et al. found that the expression of MAPLC3B in the endometrium of the RM group was significantly increased compared with that in the normal group in a controlled study of 97 infertile women (51 RM patients and 46 normal patients) who underwent *in vitro* fertilization, indicating that there is abnormal autophagy in the endometrium of the RM group (Hong et al., 2021). LC3B protein expression was upregulated in HESC cells cultured in the glutamine-deficient medium to simulate endometrial nutrient deprivation. In addition, glutamine deprivation increased the expression of MAPLC3B, ATG12, Beclin-1, GCN2, and AMPK while reducing the expression level of mTORC1. Therefore, abnormal amino acid metabolism in the endometrium can cause dysfunction of endometrial autophagy and is a key factor in the deterioration of the endometrial environment, which is closely related to the occurrence and development of RM. Pan et al. also found that RM patients were related to the activation of autophagy due to impaired Shh (Sonic Hedgehog) signaling (Pan et al., 2021). The specific mechanism is that impaired Shh signaling causes dysfunction in Shh/Gli and impacts trophoblast migration and angiogenesis, which has also been confirmed *in vitro*. Knocking down of Gli2 and supplementing with recombinant Shh in JAR cells can alleviate the inhibitory effects of the Smo antagonist cyclopamine (Cyc) and Gant 61 on trophoblastic motility and angiogenesis, mitigating the impact on trophoblast cell migration and angiogenesis, providing a novel targeted therapeutic approach for RM caused by dysregulated autophagy.

In addition, Wei et al. also found that hyperoside improved adverse pregnancy outcomes in a rat model of RM induced by (aCL)-lgG (Wei et al., 2020). The specific mechanism involved the downregulation of phosphorylated mTOR and phosphorylated p70S6 kinase (S6K) by hyperoside, as well as the downregulation of Toll-like receptor 4 (TLR4), myeloid differentiation factor 88 (MyD88), and NF-kB p-p65 expressions, enhancing the level of autophagy and exerted an anti-inflammatory effect, ultimately reducing fetal resorption. (Table 3).

**TABLE 3:** Summary of available studies associated with the treatment of pregnancy complications caused by autophagy dysfunction.

Pregnancy complications	References	Objects	Therapeutic drug	Target	Autophagy regulation
Miscarriage	Wei et al. (2020)	Rat	Hyperoside	mTOR, S6K	Promotion of autophagy
	Lu et al. (2021)	Mice	Rapamycin	mTOR	Promotion of autophagy
Intrauterine Growth Restriction	Wang et al. (2021)	Rat	Melatonin	—	Promotion of autophagy
Gestational	Liu et al. (2022)	Mice	Resveratrol	—	Promotion of autophagy
Hypertension	Hu et al. (2022)	Mice	CsA	—	Promotion of autophagy
Gestational Diabetes Mellitus	Luo et al. (2020)	Rat	Taurine	—	Inhibition of autophagy

### 4.2 The role of autophagy in premature birth

Premature birth is the live birth before completing 37 weeks of pregnancy and is the major contributing factor affecting 15 million premature babies yearly (Vogel et al., 2018; Walani, 2020; Zhao et al., 2020). Avagliano et al. conducted a comparative study on the placenta of preterm birth caused by inflammatory and non-inflammatory lesions before 34 weeks of pregnancy and found that in all preterm birth cases, trophoblast cells exhibited expression of LC3, regardless of the type of lesion (Avagliano et al., 2017a). In cases with histological inflammation, high expression of LC3 was observed in inflammatory cells composed of neutrophils. The expression of LC3 was lower in preterm birth cases with histological inflammation than in those without it. Therefore, there appears to be a close relationship between placental inflammatory lesions and autophagy expression, which could provide new therapeutic approaches for diagnosing and treating premature birth.

Cao et al. conducted a comparative study on placental samples from 40 pregnant women (divided into early preterm birth (<32 weeks), late preterm birth (32–37 weeks), and full-term birth (>37 weeks)) and showed that the level of LC3 was lower in early preterm births than in late preterm births and full-term births, while P62 was higher than in late preterm births and full-term births (Cao et al., 2016). ATG16L1 was significantly lower in early and late preterm births than in full-term births, and white blood cell counts were higher in early preterm births than in late preterm births or full-term births. *In vitro* cell experiments confirmed that the absence of ATG16L1 increased the susceptibility of fetal syncytiotrophoblasts to infection. Interestingly, the absence of ATG16L1 did not change the susceptibility of cytotrophoblasts to infection.

### 4.3 The role of autophagy in intrauterine growth restriction

Intrauterine growth restriction (IUGR) refers to the growth and developmental delay of the embryo or fetus due to changes in the intrauterine and external environments, mainly manifested as low birth weight and underdeveloped tissue and organs (Darendeliler, 2019). IUGR is not only a high-risk factor for fetal, neonatal, and infantile morbidity and mortality but is also closely related to the growth and development of the offspring after birth and can induce various diseases in adulthood (Kesavan and Devaskar, 2019).

Zhu et al. studied the effects of cadmium exposure on pregnancy by animal and cell experiments and found that fetal weight and crown-rump length of the offspring of pregnant mice in the Cd exposure group were reduced (Zhu et al., 2021). The levels of progesterone and progesterone-related enzymes (StAR and CYP11A1) in pregnant mice’s serum, placenta, and amniotic fluid in the Cd exposure group were lower than in the control group. Parkin, LC3, and TOM20 were found to be high in Cd-exposed placental tissues, and the levels of mitochondrial P4 synthesis enzymes (STAR, CYP11A1, and 3β-HSD) were significantly reduced. These results were also confirmed in cell experiments. Interestingly, the mitochondrial division inhibitor Mdivi-1 can alleviate the downregulation of P4 and the occurrence of IUGR caused by Cd exposure. Therefore, environmental Cd exposure can lead to IUGR by reducing the placenta’s progesterone level activating mitochondrial autophagy in placental tissue.

Dai et al. also conducted a comparative study of 18 normal pregnant women and 18 IUGR pregnant women (Dai et al., 2021). They found that the expression levels of Beclin-1 and MMP9 in the placenta of the IUGR group were lower than those in the normal group. In comparison, the expression levels of LC3-I, LC3-II, and ADMA (asymmetric dimethylarginine), an inhibitor of nitric oxide synthase, were higher than those in the normal group. It was revealed by *in vitro* experiments using BeWo cells (human choriocarcinoma cell line) that in cells treated with ADMA, the expression levels of mTOR and MMP9 were decreased, while the expression levels of Beclin-1 and LC3-I, LC3-II were increased, and 3-MA can inhibit the reduction effect of ADMA on MMP9. Therefore, there is excessive autophagy induced by ADMA in the placenta of the IUGR group, which can induce the occurrence of IUGR by weakening the invasion ability of trophoblasts. The above studies indicate that exposure to adverse factors and dysregulation of placental functional proteins are also closely related to the occurrence and development of IUGR.

In addition, Wang et al. confirmed that the enriched environment (EE) and melatonin (MEL) could improve the adverse effects of IUGR on the development of offspring rats by activating the IGF-1/IGFBP1 and IGF-1/mTOR/S6K1/4EBP1 signaling pathways and inhibiting autophagy (Wang et al., 2021). Initially, they established IUGR rat models using a low-protein diet and divided them into a control group, model group, and MEL + EE group. Subsequent examination of the offspring fetuses revealed that, compared to the model group, the MEL + EE group showed an increase in the number of fetal rats and a decrease in the expression of Beclin-1 and LC3B in the offspring liver, providing an entirely new approach to the treatment of IUGR (Table 3).

### 4.4 The role of autophagy in gestational hypertension

Gestational hypertension is one of the most serious complications during pregnancy, with about 2%–8% of pregnant women worldwide having pre-eclampsia (Obstet Gynecol, 2019; Tsakiridis et al., 2021). There exists a multitude of intricate factors contributing to the development of gestational hypertension. Recent investigations have revealed a strong correlation between gestational hypertension and the impairment of placental autophagy (Wilkerson and Ogunbodede, 2019). Sharma et al. found that lacking ATG7 in the placenta may lead to gestational hypertension in mice, mainly manifested by an increase in P62 expression, a decrease in the invasion of placental trophoblasts, and an increase in cell apoptosis, resulting in pre-eclampsia-like symptoms such as elevated blood pressure, proteinuria, and fetal growth restriction in pregnant mice (Sharma, 2018).

In addition, Lee et al. found that the deficiency of ATG7 can lead to autophagy dysfunction in uterine stromal cells, myometrial cells, and vascular smooth muscle cells but not in endothelial cells (Lee et al., 2021). The specific mechanism is that the lack of Atg7 can cause autophagic flux to be blocked by accumulating a large amount of SQSTM1/P62 and MAP1LC3B/LC3B in the uterus. This change is more significant in one-year-old mice and positively correlated with the age of the mice. Abnormal regulation of autophagy in the uterus of mice can also lead to an increase in the expression of VEGFA, VSMCs, and NOS (NOS1, NOS2) in uterine blood vessels, disruption of inter-endothelial cell junction and causing pathological changes such as high permeability and high osmosis in uterine blood vessels. This change persists in pregnant mice. The nitric oxide synthase inhibitor L-NAME can inhibit NOS to reduce vascular permeability. Therefore, the dysregulation of autophagy in the uterus can induce pathological changes in uterine blood vessels, thereby changing the placental blood supply and nutrient supply, leading to gestational hypertension-related diseases.

On the other hand, there is increasing attention on the treatment of gestational hypertension caused by abnormal regulation of autophagy. Liu et al. found that supplementation of SIRT1/SIRT agonists (resveratrol) in SIRT1-deficient mice reversed the symptoms of pre-eclampsia in mice (Liu et al., 2022). The specific mechanism involves SIRT1 activating autophagy-related factors (such as TFEB, LC3B, Beclin-1, and P62) in mouse placenta through deacetylation, thereby improving the level of autophagy in trophoblast cells and alleviating the adverse effects caused by impaired placental autophagy. (Table 3). Hu et al. also found that supplementation of Cyclosporin A (CsA) in the nitro-l-arginine methyl ester (l-NAME)-induced preeclamptic mouse model, as well as in an *in vitro* hypoxia-reoxygenation model, alleviated placental necrosis and senescence, and reduced cellular damage caused by hypoxia-reoxygenation by upregulating the expression of autophagy proteins (Hu et al., 2022). This was done to improve adverse pregnancy outcomes caused by preeclampsia. (Table 3).

### 4.5 The role of autophagy in gestational diabetes mellitus

Gestational diabetes mellitus (GDM) is a temporary form of diabetes caused by insulin resistance and pancreatic beta-cell dysfunction during pregnancy (Alejandro et al., 2020). Although there are various treatments available for GDM, its incidence remains high. Current studies on the changes in autophagy in the placenta of GDM pregnant women have produced conflicting results. Avagliano et al. found that in the placenta of GDM pregnant women, Beclin-1 was significantly reduced, and LC3-II protein accumulated due to inhibition of autophagosome degradation (Avagliano et al., 2017b). Interestingly, the researchers also found an increase in the number of pancreatic beta-cells and LC3 expression levels in stillborn fetuses of GDM patients, which may be related to the upregulation of autophagy caused by insulin resistance. Wang et al. found that enhanced autophagy in the placenta of GDM pregnant women was closely associated with the upregulation of death-associated protein kinase-3 (DAPK3) (Wang et al., 2020). High-glucose treatment of the HTR8/SVneo trophoblast cell *in vitro* led to upregulation of DAPK3 expression while knocking down DAPK3 inhibited autophagic flux by blocking the formation of the STX17-SNAP29-VAMP8 complex, leading to increased expression of LC3-II, ATG5, and P62, and promoting invasion of trophoblast cells. Li et al. also concluded that the placenta weight and the expression of autophagy-related genes ATG7 and LC3B were higher in placental tissue of GDM pregnant women than in normal pregnant women, indicating an increase in autophagic flux (Li et al., 2020).

Hung et al. performed a more refined grouping of GDM pregnant women: those with large-for-gestational-age (LGA) infants and those with appropriate-for-gestational-age (AGA) infants (Hung et al., 2020). Analysis showed that the placental weight of GDM pregnant women with LGA significantly increased compared to normal pregnant women. In addition, the expression of Beclin-1 and DRAM was decreased, while P62 was significantly increased, and both cell autophagy and apoptosis were decreased, but cell proliferation was increased. However, there were no changes in placental weight or corresponding proteins in GDM pregnant women with AGA infants. *In vitro* experiments showed that increasing glucose concentration in the culture medium of trophoblast cells simulated the GDM environment, resulting in decreased expression of ATG5, Beclin-1, LC3B-II, and P62, consistent with the trend of changes in placental tissue of GDM pregnant women with LGA.

Currently, there exists inconsistency in the findings regarding alterations in autophagic flux within the placenta of pregnant women with GDM and trophoblast cells treated with high glucose. To establish the integrity of these results, it is imperative to conduct a greater number of clinical samples and delve into more comprehensive mechanistic investigations. In addition, the treatment of gestational diabetes caused by abnormal regulation of autophagy has also attracted more and more attention. Luo et al. established a GDM rat model by injecting 1% streptozotocin and a high-fat diet 1 week before pregnancy and studied the livers of 8-week-old offspring rats to explore the effects of GDM rats on offspring liver damage (Luo et al., 2020). Compared with normal offspring rats, the expression levels of LCII and Beclin-1 in the offspring livers of the GDM group were significantly increased, and the number of autophagosomes in hepatocytes, hepatocyte apoptosis, and hepatocyte edema were also increased; Bcl2, p62, and PPARγ protein expressions were significantly decreased. Interestingly, taurine supplementation can treat this injury by reducing the expression of LCII, Beclin-1, Bax, and cleaved-caspase3/caspase3. (Table 3).

### 4.6 The role of autophagy in gestational obesity

Several studies have shown that maternal obesity can cause significant changes in insulin resistance, resulting in high levels of insulin, leptin, IGF-1, lipids, and some pro-inflammatory factors in the plasma (Kelly et al., 2020; Davis and Mire, 2021; Fowden et al., 2021). High levels of insulin, leptin, IGF-1, and adiponectin can activate the mTOR signaling pathway in the placenta, affecting protein synthesis, mitochondrial function, and nutrient transport, which increases the risk of disease in both the mother and fetus. Simon et al. extracted trophoblast cells from chorionic villi of term placenta and treated with TNF-α to simulate the obese intrauterine inflammatory environment (Simon et al., 2017). The cells treated with TNF-α exhibited multinucleated cell clusters and syncytialization changes, while the villous trophoblast of male fetuses of obese mothers showed autophagosome formation and autophagy activation. However, in the villous trophoblast of female fetuses, autophagic flux did not change significantly due to the upregulation of Rubicon, a negative regulator of autophagy that can prevent autophagosome-lysosome fusion. This compensatory protective mechanism against TNF-α-mediated inflammation could provide a new therapeutic target for regulating autophagy.

Upadhyay et al. found that in obese pregnant rats induced by high-fat feeding, both LC3-II and P62 are elevated in the placenta, indicating that autophagolysosome fusion defects cause a late-stage block of placental autophagy flux, leading to increased apoptosis (Upadhyay et al., 2019). Time-restricted feeding improves autophagy defects and reduces placental inflammation and ERS caused by high-fat feeding by restoring ATG14L expression. Additionally, Diceglie et al. conducted a comparative study on pregnant women with GDM combined with obesity, normal-body weight pregnant women, and obese pregnant women without complications (Diceglie et al., 2021). They found that in obese pregnant women without complications, the expression of antioxidant genes CAT, GPX1, and GSS is reduced, and ULK1, a core factor in the initial stage of macroautophagy, is upregulated, while the regulatory factor of CMA, PHLPP1, shows fetal sex-dependent differences; it was downregulated in the female fetus. In obese women with GDM, PHLPP1 expression increases in both fetal genders' placentas. These studies indicate that lipid metabolism and fetal sex are closely related to changes in autophagy in the placenta of obese pregnant women.

## 5 Conclusion

**FIGURE 2:**
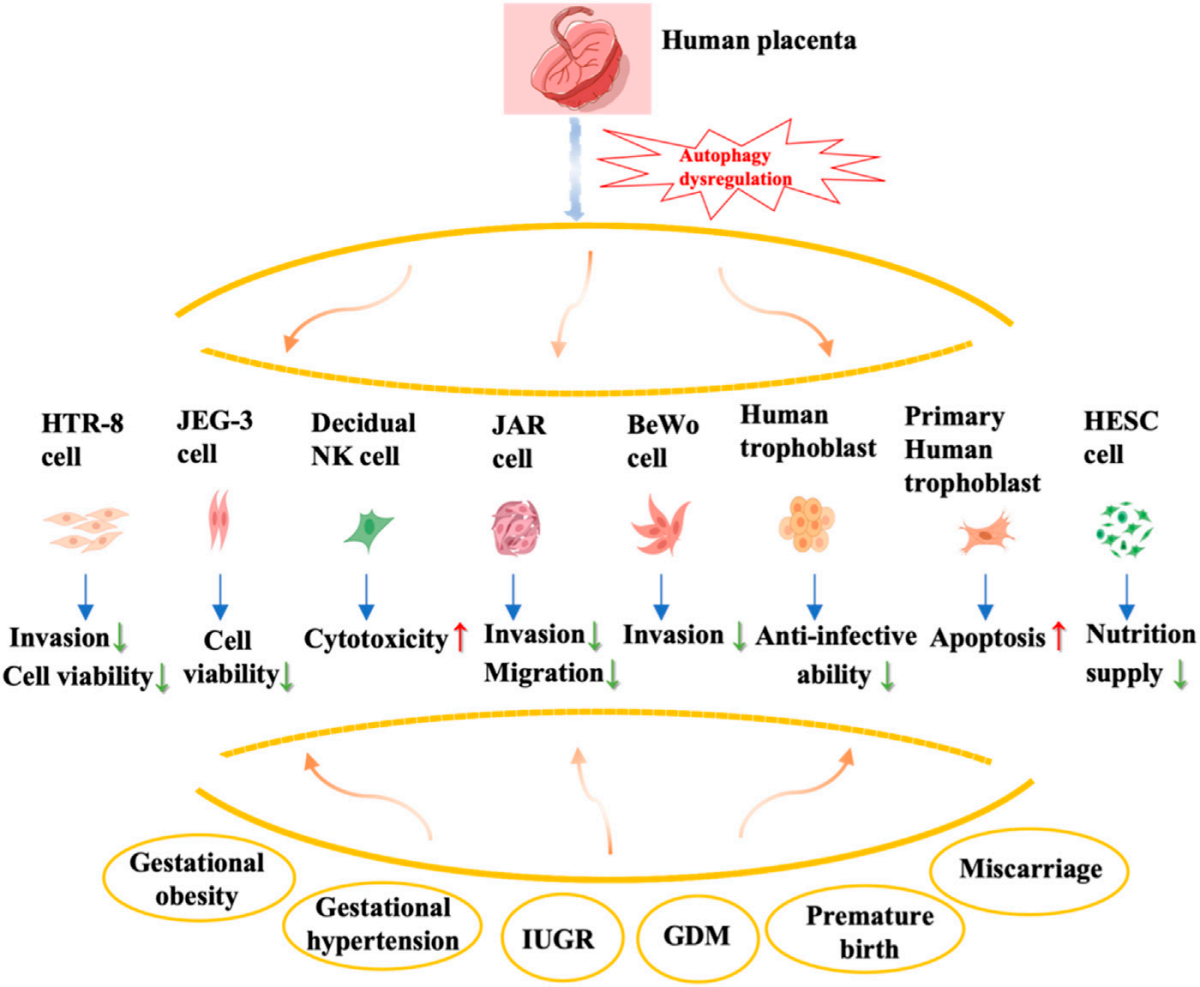
Summary of autophagy dysregulation in the placenta and associated complications during pregnancy. The effects of autophagy dysregulation in various types of placental cells, including trophoblast cells (primary cytotrophoblast, HTR-8), choriocarcinoma cell lines (JEG-3, JAR, BeWo), human endometrial stromal cells, and decidual natural killer cells, on their biological behavior is shown in this figure.

This review provides a comprehensive overview of the alterations and impacts of autophagy regulation in the placenta during various prevalent pregnancy complications, such as miscarriage, preterm birth, gestational obesity, and gestational diabetes mellitus (Figure 2). Nevertheless, existing studies predominantly concentrate on autophagy in trophoblast cells, while the understanding of autophagy modifications in other placental cell types, including decidua macrophages and T cells, remains elusive and necessitates additional investigation. Autophagy serves as a dynamic and crucial protective mechanism for preserving organismal stability and counteracting external threats. However, an excessive occurrence of autophagy can potentially result in autophagic cell death, thereby leading to misconceptions regarding the outcomes and regulatory mechanisms.

Autophagy plays a significant role in the pathophysiology of diverse pregnancy complications; nevertheless, the lack of precise estimation of alterations in autophagy flux within placental tissue hinders a clear understanding of the association between autophagy and these complications. Future research directions primarily involve investigating the potential of autophagy-related molecules as predictive biomarkers for pregnancy complications and expanding clinical sample research to elucidate the abnormal autophagy mechanism for the purpose of identifying diagnostic markers. However, challenges remain in determining the sampling source and assessing the diagnostic efficacy of these markers. Additionally, exploring potential treatments for pregnancy complications through targeting the autophagy pathway, such as utilizing autophagy inhibitors or activators, is another important avenue for future investigation. For instance, rapamycin, an MTOR mTOR inhibitor, has been shown to effectively induce autophagy in the trophoblast, thereby reducing NK cytotoxicity. Additionally, it promotes deciduous stromal autophagy and NK residency in the decidua, ultimately preventing spontaneous abortion (Lu et al., 2021) (Table 3). By elucidating the role of this ancient self-defense mechanism in the pathogenesis of pregnancy complications, novel approaches can be developed for the early detection and prevention of such complications.
